# Evaluation of Bone Regeneration by Sustained Release of Bone Morphogenetic Protein from Porous Polyetheretherketone

**DOI:** 10.3390/ijms27209337

**Published:** 2026-10-21

**Authors:** Yuka Shiotani, Jun-ichiro Jo, Sara Takasugi, Takeryo Adachi, Kenichirou Yasui, Yoshiya Hashimoto, Aki Nishiura

**Affiliations:** 1 Department of Orthodontics, Osaka Dental University, 8-1 Kuzuhahanazonocho, Hirakata 573-1121, Osaka, Japan; shiotani-y@cc.osaka-dent.ac.jp (Y.S.); takeryo.adachi@gmail.com (T.A.); yasui-k@cc.osaka-dent.ac.jp (K.Y.); nishiura@cc.osaka-dent.ac.jp (A.N.); 2 Department of Biomaterials, Osaka Dental University, 8-1 Kuzuhahanazonocho, Hirakata 573-1121, Osaka, Japan; yoshiya@cc.osaka-dent.ac.jp

**Keywords:** polyetheretherketone (PEEK), bone morphogenetic protein-2 (BMP-2), gelatin hydrogel, sustained release, bone regeneration

## Abstract

Owing to its excellent mechanical properties and biocompatibility, polyetheretherketone (PEEK) is a promising biomaterial in dental and medical fields. However, PEEK lacks osteoconductive and osteoinductive properties for efficient bone regeneration. In the present study, we aimed to develop a porous PEEK scaffold integrating gelatin hydrogel as a carrier for the sustained delivery of bone morphogenetic protein-2 (BMP-2) and evaluate its bone regeneration potential. The pores in the three-dimensional printed porous PEEK were filled with an aqueous solution of gelatin, followed by freeze-drying and dehydrothermal crosslinking to obtain a porous PEEK scaffold with gelatin hydrogel (Gel-PEEK). BMP-2 dissolved in phosphate-buffered saline was impregnated into Gel-PEEK to obtain Gel-PEEK incorporating BMP-2 (BMP-2@Gel-PEEK). After confirming the sustained release of BMP-2 from Gel-PEEK in vitro, the bone regeneration potential of BMP-2@Gel-PEEK was evaluated using a model of critical-sized calvarial defects in rats. Micro-computed tomography and histological analyses demonstrated augmented bone regeneration by BMP-2@Gel-PEEK compared with PEEK only and Gel-PEEK without incorporating BMP-2. These findings suggest that through the sustained release of BMP-2, the porous PEEK with gelatin hydrogel is a promising platform for bone regeneration.

## 1. Introduction

Bone defects caused by disease, trauma, or tumor resection often necessitate maxillofacial bone reconstruction. Grafting with autologous bone is currently considered to be the gold standard for bone reconstruction [1,2]. However, issues such as complications at the donor site and the limited availability of autologous bone present significant clinical challenges, particularly in cases requiring large-volume bone regeneration [3]. Under certain circumstances, novel biomaterials, technologies, and methodologies are crucial for bone reconstruction beyond autologous bone grafting. Numerous studies have demonstrated the importance of tissue engineering in the efficient introduction of tissue regeneration using stem cells, scaffolds, and growth factors [4,5]. Scaffolds contribute to the maintenance or augmentation of biological activities of stem cells [6,7,8]. Three-dimensional (3D) scaffolds have been actively developed to structurally and biologically mimic the natural interaction of cells with the extracellular matrix [9,10,11].

Polyetheretherketone (PEEK) is a super-engineering plastic that exhibits thermal and chemical stability as well as superior mechanical properties and has been used in the engineering field [12]. Recently, PEEK has been used as a biomaterial in dental and medical fields, such as dental implantology, prosthodontics, and alveolar bone reconstruction owing to the excellent biocompatibility and elastic modulus comparable to that of natural bone [13,14,15]. In addition, PEEK can be fabricated three-dimensionally by printing owing to its thermoplastic property [16,17]. However, PEEK itself lacks osteoconductive and osteoinductive properties; there is still room for further development aiming at biologically efficient bone reconstruction.

Bone morphogenetic protein-2 (BMP-2) is a robust osteoinductive growth factor that promotes osteoblast differentiation and bone formation [18,19,20]. Despite its potential, the biological activity is limited after in vivo local administration of BMP-2 in solution form, because BMP-2 rapidly disappears from the administration site. Therefore, the development of a delivery system is essential for the safe and effective clinical application of BMP-2. Various biomaterials have been developed as a carrier for BMP-2 controlled release [21,22,23,24]. Among them, gelatin hydrogel possesses excellent biocompatibility and biodegradability and has been widely investigated as a sustained-release carrier for bioactive molecules [25,26]. We prepared a bioactive PEEK substrate by combining it with gelatin hydrogel incorporating BMP-2 and demonstrated promoted osteogenic differentiation in vitro [27].

In this study, we developed a bioactive porous PEEK scaffold with sustained BMP-2 release by integrating gelatin hydrogel into three-dimensionally fabricated porous PEEK. The originality of this study lies in the attempt to introduce the sustained release technology to a bioinert 3D porous PEEK scaffold. We explored the characteristics of porous PEEK with gelatin incorporating BMP-2 by evaluating the BMP-2 release behavior in vitro and bone regeneration potential using a rat calvarial defect model.

## 2. Results

### 2.1. In Vitro Evaluation of Porous PEEK with Gelatin Hydrogel Incorporating BMP-2 (BMP-2@Gel-PEEK)

**Figure 1:**
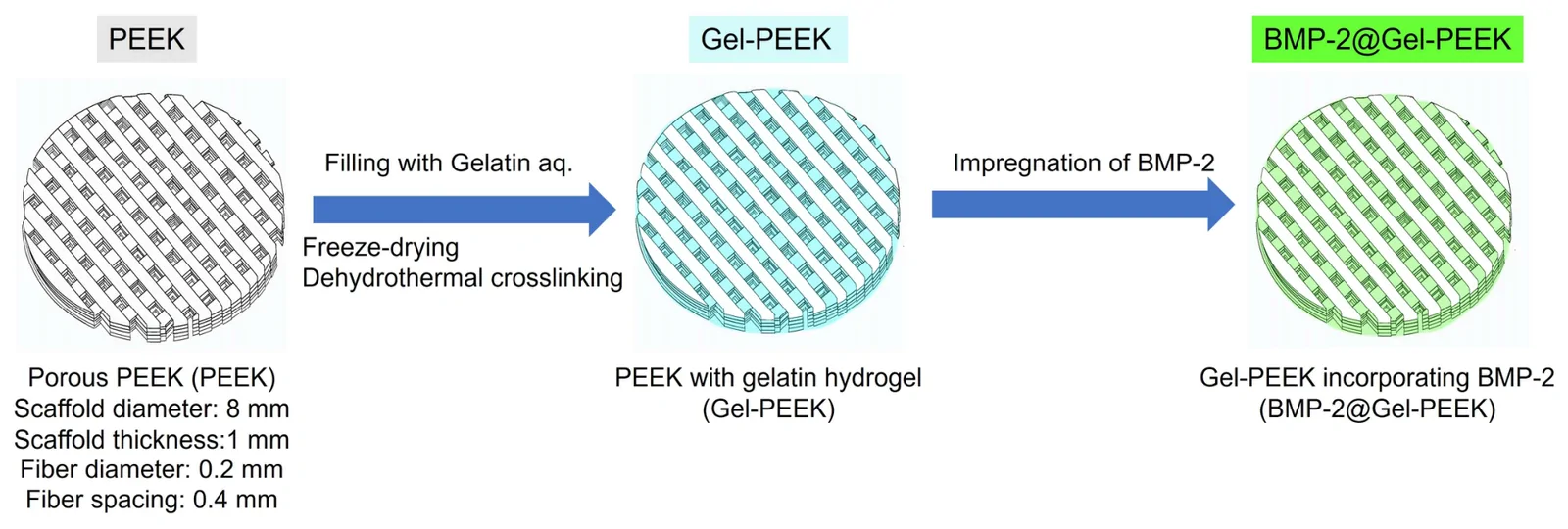
Preparation scheme of porous PEEK with gelatin hydrogel incorporating BMP-2.

Figure 1 shows the preparation scheme of BMP-2@Gel-PEEK. Porous PEEK with gelatin hydrogel (Gel-PEEK) was prepared by filling the pores in porous PEEK with a gelatin aqueous solution, followed by freeze-drying and dehydrothermal crosslinking. BMP-2@Gel-PEEK was prepared by impregnating BMP-2 into Gel-PEEK.

**Figure 2:**
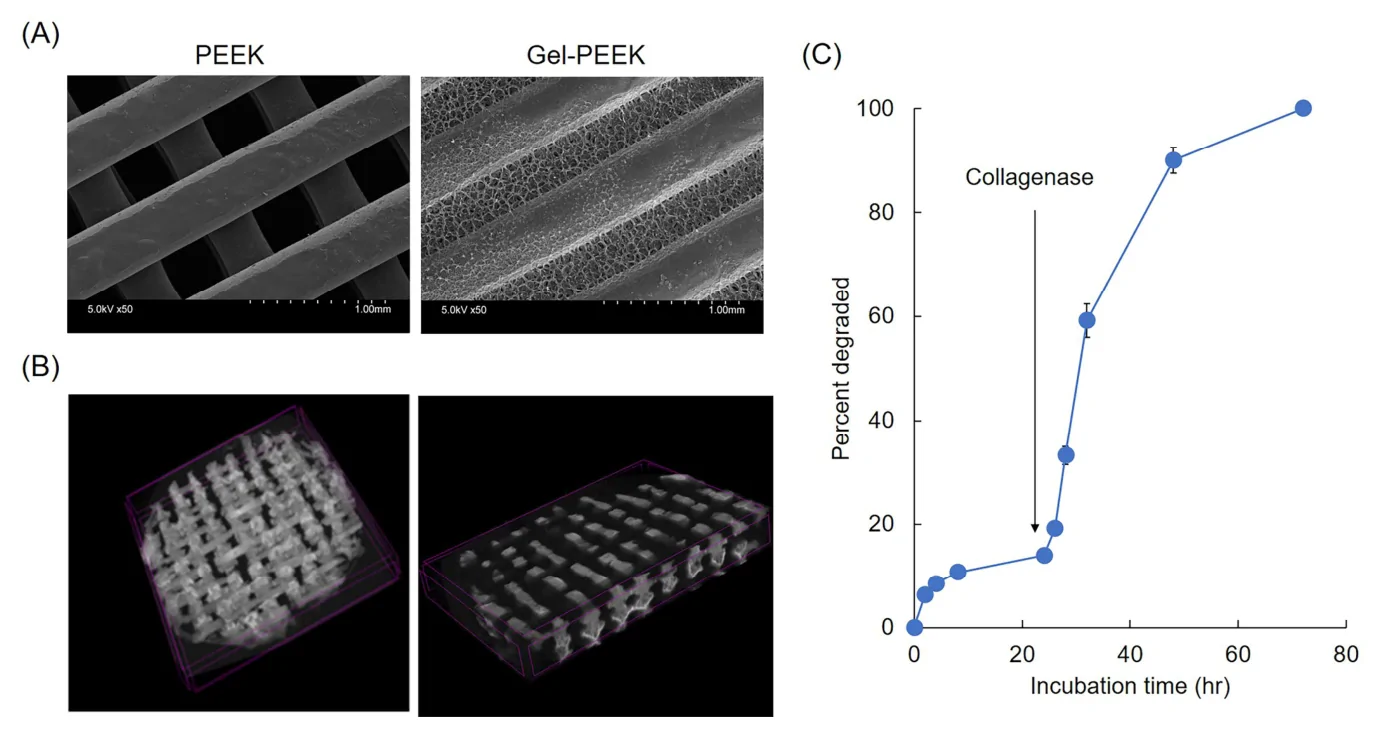
Characterization of porous PEEK with gelatin hydrogel (Gel-PEEK). (**A**) SEM images of porous PEEK and Gel-PEEK. (**B**) Representative three-dimensional μCT image of Gel-PEEK. Gelatin hydrogel was visualized by staining with iodine, a CT contrast agent. (**C**) Degradation profile of gelatin hydrogel in porous PEEK. Gel-PEEK was incubated in PBS for the initial 24 h and in PBS containing collagenase.

The microstructure of PEEK or Gel-PEEK scaffolds was observed through SEM (Figure 2A). The PEEK scaffold had a porous structure with regular grid patterns. The pore size was approximately 400 μm, which was consistent with that designed by 3D fabrication. In the Gel-PEEK scaffold, gelatin hydrogel is incorporated into the pores of the porous PEEK scaffold. The 3D micro-computed tomography (μCT) image of the Gel-PEEK scaffold indicated the visualization of gelatin hydrogel throughout the pores in the porous PEEK scaffold (Figure 2B). These results clearly indicate that the Gel-PEEK scaffold, where almost all of the pores in the PEEK scaffold are filled with a gelatin hydrogel, could be prepared by adding a gelatin aqueous solution into the pores in the PEEK scaffold.

The degradation behavior of gelatin hydrogel in the Gel-PEEK scaffold was evaluated by measuring the amount of gelatin eluted after soaking in phosphate-buffered saline (PBS) containing collagenase (Figure 2C). Although the degradation of gelatin hydrogel without the addition of collagenase was limited to approximately 15%, the degradation of gelatin hydrogel was accelerated by the addition of collagenase.

**Figure 3:**
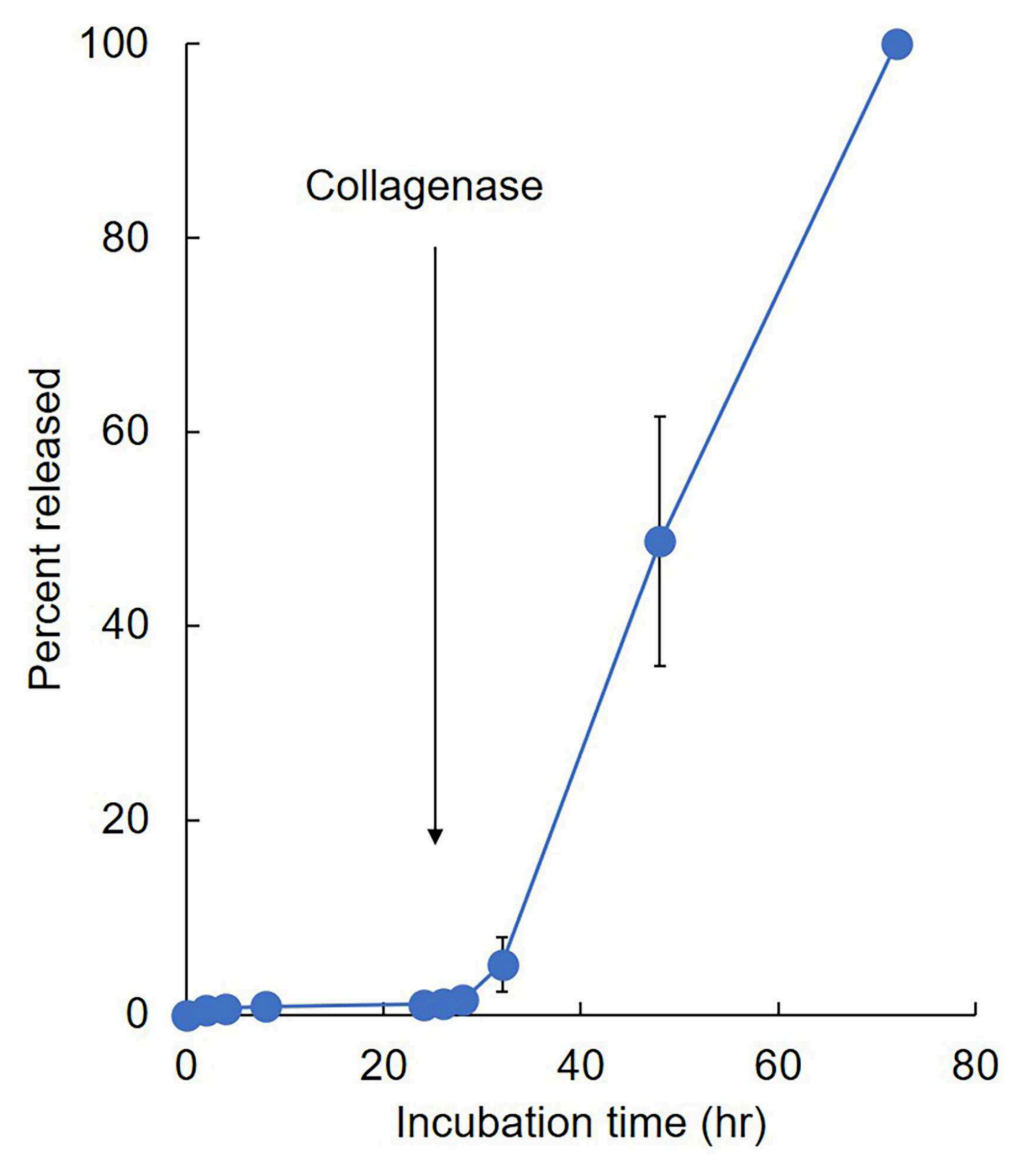
Profile of BMP-2 release from Gel-PEEK. BMP-2@Gel-PEEK was incubated in PBS for the initial 24 h and in PBS containing collagenase.

The release profile of BMP-2 from Gel-PEEK was investigated by measuring the amount of BMP-2 under the same conditions as the degradation test (Figure 3). The BMP-2 release was inhibited until the addition of collagenase, after which it was observed in a sustained manner. These results demonstrate that the combination of gelatin hydrogel with the porous PEEK scaffold enabled the sustained release of BMP-2.

### 2.2. In Vivo Evaluation of BMP-2@Gel-PEEK

**Figure 4:**
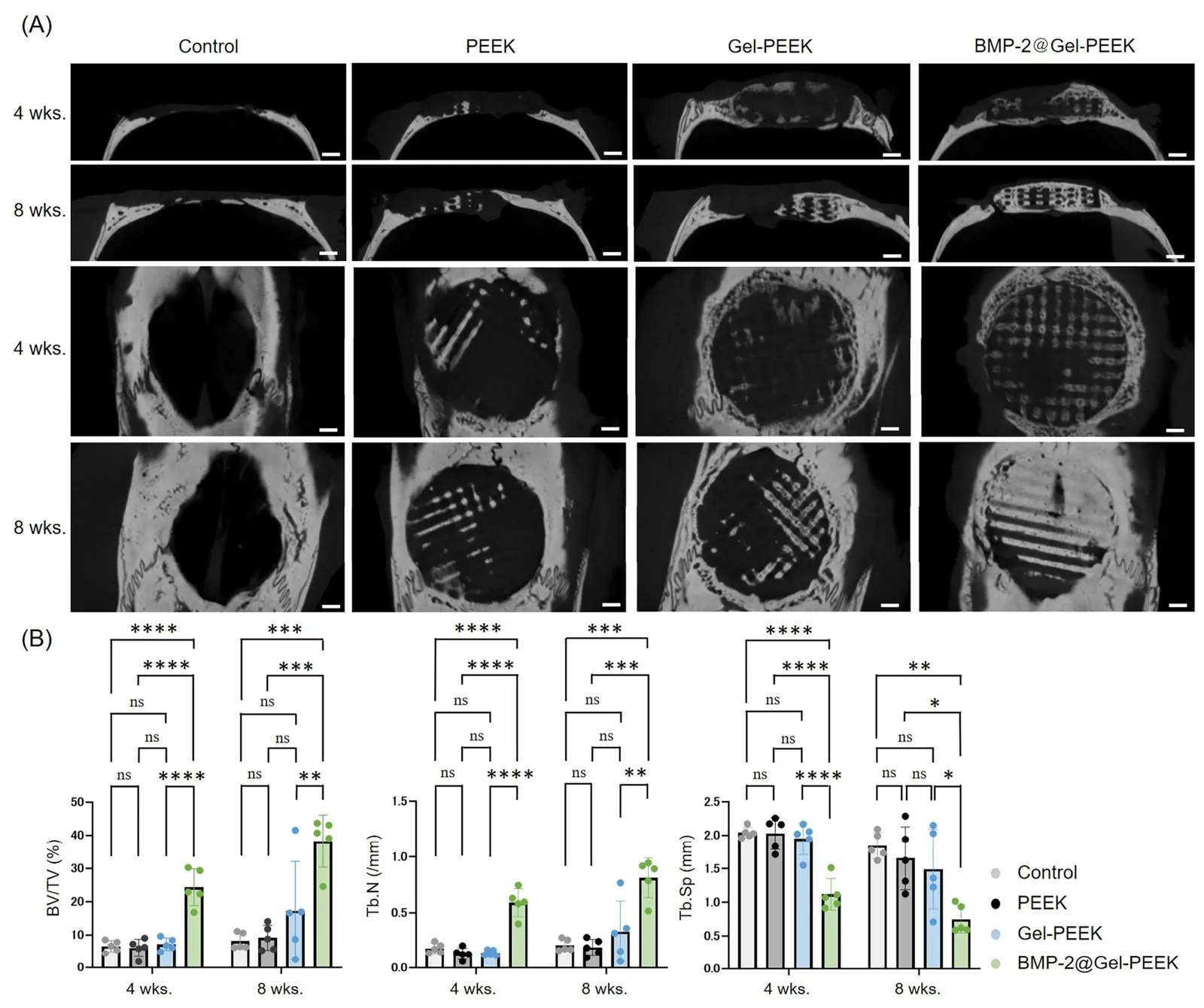
Evaluation of bone formation for critical-sized bone defects in rats 4 and 8 weeks after implantation of each scaffold by X-ray μCT. Bone defects without scaffold implantation were set as the control. (**A**) Representative coronal (upper) and axial (bottom) plane images of the defect site. The scale bar is 1 mm. (**B**) Quantitative values obtained from μCT analysis: ratio of bone volume to total volume (BV/TV, %), trabecular number (Tb.N, 1/mm), and trabecular separation (Tb.Sp, mm). *, **, ***, and **** indicate *p* < 0.05, 0.01, 0.001, and 0.0001, respectively. The “ns” indicates no statistically significant difference.

The bone formation for critical-sized bone defects in rats 4 and 8 weeks after the implantation of each scaffold was first evaluated using X-ray μCT (Figure 4A). Almost no bone formation was observed in the control group. In the PEEK, Gel-PEEK, and BMP-2@Gel-PEEK groups, bone formation was observed at 4 weeks post-implantation; however, the extents varied. In the PEEK and Gel-PEEK groups, bone formation was detected mainly at the margins of the defect, whereas in the BMP-2@Gel-PEEK group, more prominent bone formation was observed throughout the porous region of the scaffold. At 8 weeks post-implantation, the bone formation proceeded in all groups. In particular, in the BMP-2@Gel-PEEK group, bone formation was observed even outside the scaffold.

The bone parameters, including the volume ratio of bone to tissue (BV/TV), the trabecular number (Tb.N), and the trabecular separation (Tb.Sp), were analyzed after μCT acquisition (Figure 4B). The values of BV/TV and Tb.N in the BMP-2@Gel-PEEK group were significantly higher than those in other groups at both 4 and 8 weeks after implantation. Conversely, the Tb.Sp in the BMP-2@Gel-PEEK group had the lowest values at both 4 and 8 weeks post-implantation, with a significant difference compared with those in other groups. These results indicate an increase in new bone mass as well as densification of the trabecular bone structure, suggesting the formation of bone tissue with denser trabecular architecture in the BMP-2@Gel-PEEK group.

**Figure 5:**
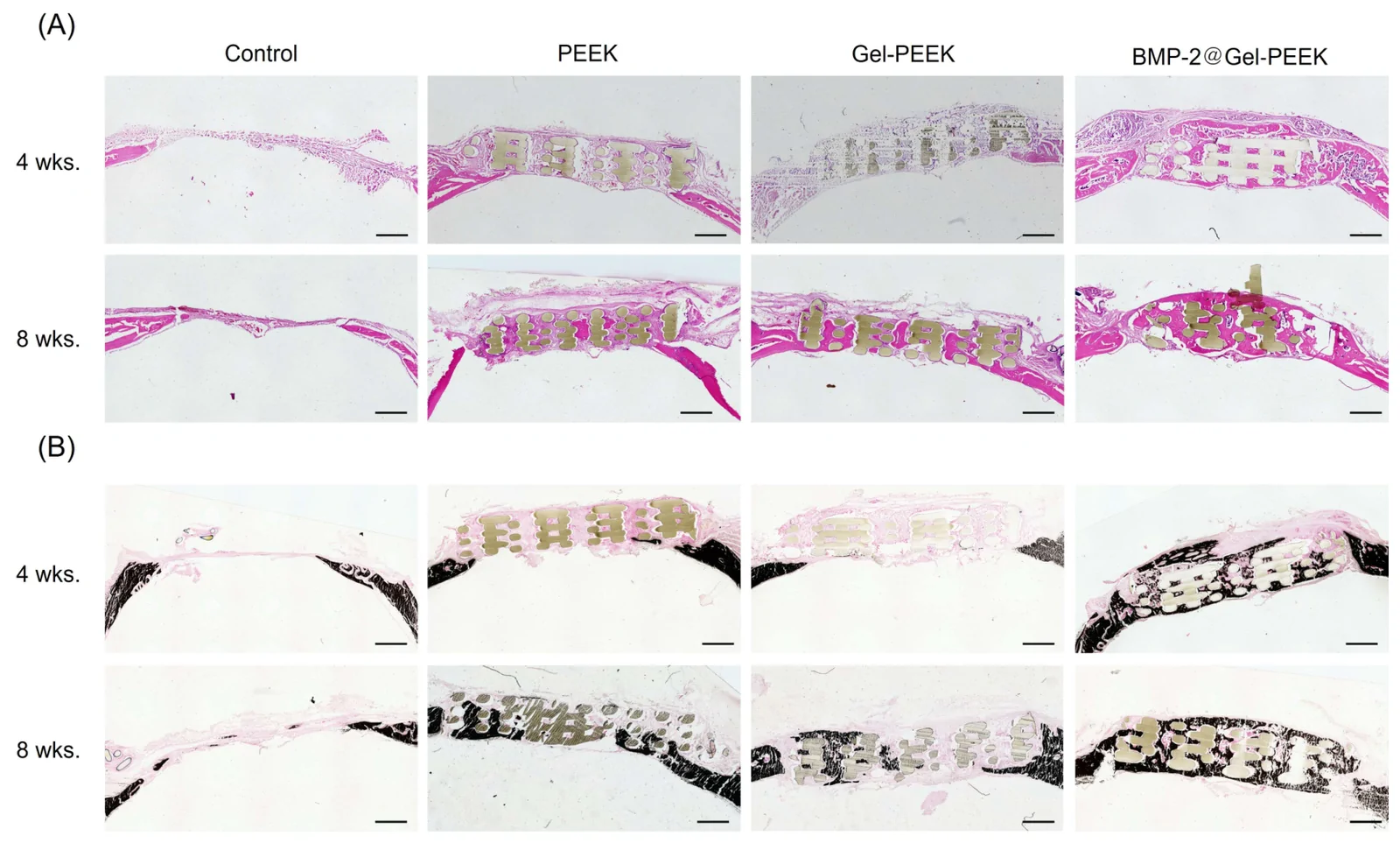
H&E staining (**A**) and vK staining (**B**) images of tissue around bone defect 4 and 8 weeks after implantation of each scaffold. Bone defects without scaffold implantation were set as the control. The scale bar is 1 mm.

**Figure 6:**
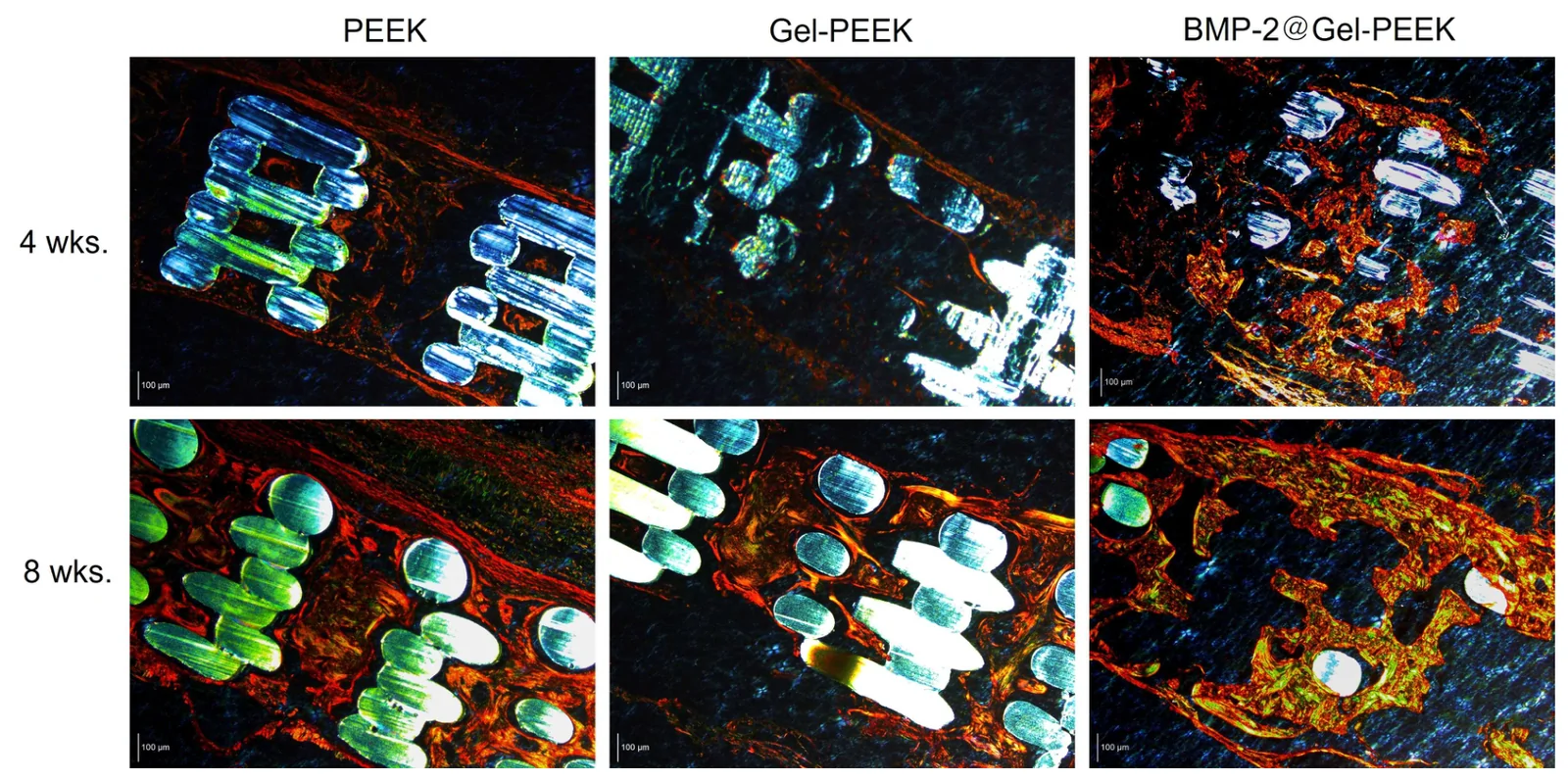
SR staining images of the interior of each scaffold by polarized microscopy at 4 and 8 weeks post-implantation. The scale bar is 100 μm.

The post-implantation tissues were histologically evaluated through several methods. The hematoxylin and eosin (H&E) staining explores the general tissue condition (Figure 5A). The H&E staining revealed no signs of infection or inflammation in all groups at any time point. At 4 weeks, prominent tissue formation covering the defect site was observed in the BMP-2@Gel-PEEK group, whereas only limited tissue formation was detected in the control group. By 8 weeks, tissue formation had progressed in all groups. Furthermore, tissue bridging across the defect was observed in the PEEK, Gel-PEEK, and BMP-2@Gel-PEEK groups. The von Kossa (vK) staining visualizes the calcification during bone formation (Figure 5B). The vK staining revealed limited, low-density calcification in both the PEEK and Gel-PEEK groups at 4 weeks, and the extent of calcification increased by week 8. Conversely, extensive and high-density calcification was observed in the BMP-2@Gel-PEEK group as early as 4 weeks. The Sirius red (SR) staining observed by polarized light microscopy revealed the state of collagen fibers produced during bone formation (Figure 6). In the BMP-2@Gel-PEEK group, abundant collagen fiber formation was observed at 4 weeks post-implantation, while the fiber structure became more continuous at 8 weeks post-implantation.

## 3. Discussion

In this study, we integrated a gelatin hydrogel into the pores of porous PEEK to create a bioactive scaffold capable of sustained release of BMP-2. The gelatin hydrogel in porous PEEK released BMP-2 upon enzymatic degradation, and the BMP-2@Gel-PEEK group exhibited the highest bone regeneration in a rat parietal bone defect model. These results demonstrate that a BMP-2 sustained release system utilizing a gelatin hydrogel effectively enhances the osteogenic potential of porous PEEK.

The sustained release of BMP-2 depended on the enzymatic degradation of the gelatin hydrogel (Figure 2 and Figure 3). Various materials for hydrogel preparation have been used as one of the most feasible carriers for sustained release [28,29,30,31]. Among hydrogel materials, gelatin is derived from denaturation of collagen present in the extracellular matrix and is widely used as a sustained-release carrier for bioactive factors because it undergoes enzymatic degradation in vivo [32,33]. BMP-2 was retained within the gelatin matrix and was gradually released, associated with matrix degradation. Such an enzyme-responsive release mechanism has the potential to suppress initial burst release and provide a sustained supply of growth factors.

In the present study, the release profile of BMP-2 was evaluated assuming that all of the BMP-2 was loaded into the gelatin hydrogel due to the visual confirmation of BMP-2 sorption. The quantitative evaluation of loading efficiency and cumulative release will clearly characterize the sustained release property of the gelatin hydrogel. In addition, the in vitro release study aimed to evaluate the capability of the gelatin hydrogel in the porous PEEK scaffoldto provide the sustained release of BMP-2. Therefore, the concentration of collagenase used for the in vitro release experiment was different from the in vivo condition, and the in vitro release experiment does not adequately reproduce the in vivo environment. In the present study, different amounts of BMP-2 were used between in vitro and in vivo studies. Considering the detection sensitivity and cost performance, a small amount of BMP-2 (1 μg) was selected in the in vitro study. On the other hand, the BMP-2 amount (5 μg) was selected for the in vivo experiment based on previous research [34,35].

The μCT image and analysis (Figure 4) revealed that the BMP-2@Gel-PEEK group exhibited the most pronounced bone formation. BMP-2 is a powerful growth factor with osteoinductive properties that induces the differentiation of undifferentiated mesenchymal cells into osteoblasts. The present study lacks the evaluation of BMP-2 bioactivity released from gelatin hydrogel within the porous PEEK scaffold. However, previous research indicated the promoted osteoblastic differentiation of mesenchymal stem cells by the BMP release from gelatin hydrogel attached to a PEEK substrate [27]. Therefore, BMP-2 released from gelatin hydrogel within the porous PEEK scaffold is likely to display bioactivity. In the BMP-2@Gel-PEEK group, osteogenesis of mesenchymal stem cells surrounding the implantation site was likely to be enhanced by the BMP-2 sustained release. In the PEEK group, bone ingrowth into the pores was also observed over time, suggesting that the porous structure itself possesses osteoconductivity. This point agrees with a previous study using a similar porous structure of polycaprolactone [36]. However, despite the suppression of early bone formation in the Gel-PEEK group, bone formation was observed within the pores 8 weeks after implantation. This result might be attributed to the gelatin hydrogel physically restricting cell infiltration during the early stages of implantation, and cell and vascular invasion occurred as the hydrogel subsequently degraded. The histological staining for remaining gelatin hydrogel and immunohistochemical staining for CD31/endomucin and osteogenic markers, which were missing in the present study, will reveal the mechanism in detail.

The time points for evaluation of the animal experiment were limited to 4 and 8 weeks after scaffold implantation. These conditions were selected based on previous research on bone formation in the rat calvarial defect using BMP-2 [34,37]. Long-term evaluation will be necessary to evaluate the potential of the scaffold for bone regeneration. In addition, this study lacks a free BMP-2 group as a control in the animal experiment. Several papers have already demonstrated the immediate diffusion and disappearance of free BMP-2 from the injection site and the sustained release of BMP-2 by gelatin hydrogel [25,38]. An animal experiment was performed without control of free BMP-2 based on these results.

Histological evaluation (Figure 5 and Figure 6) suggested the mechanism of the best bone formation by the BMP-2@Gel-PEEK group. The BMP-2@Gel-PEEK group exhibited calcification and extracellular matrix formation by sustained release of BMP-2 from gelatin hydrogel integrated in the pores of the porous PEEK scaffold, which was consistent with the μCT result (Figure 4). Because the matrix calcification occurs during osteogenic differentiation, released BMP-2 plausibly interacted with cells infiltrated into the pores of the porous PEEK scaffold. The present study lacks a quantitative evaluation of histology. The quantitative evaluation will elucidate the detailed mechanism underlying bone formation by the BMP-2@Gel-PEEK and clarify the quantity and quality of formed bone.

The BMP-2@Gel-PEEK is a promising scaffold for bone formation that combines the excellent mechanical properties of PEEK with the sustained release capability of gelatin hydrogel. A comparison of osteogenic properties with autologous or allogeneic grafts will reveal the practical applicability of the BMP-2@Gel-PEEK scaffold in bone formation. Currently, titanium plates and meshes are widely used for maxillofacial reconstruction because of their excellent mechanical properties and ability to maintain the space required for bone regeneration [39,40]. Furthermore, recent advances in technologies of computer-aided design/computer-aided manufacturing and additive manufacturing have enabled the precise fabrication of patient-specific titanium implants based on individual CT data [41,42,43,44]. However, these devices primarily provide mechanical support and generally require additional bone graft materials or biological factors to facilitate bone regeneration. In contrast, the Gel-PEEK scaffold developed in this study provides both structural support and sustained BMP-2 delivery. Therefore, patient-specific porous PEEK scaffolds combining a BMP-2 delivery system may represent a novel strategy for bone reconstruction by simultaneously restoring defect morphology and promoting bone regeneration.

In the future, BMP-2@Gel-PEEK is expected to be used as an alternative material for jawbone reconstruction and alveolar cleft bone grafting. However, this study involved a short-term evaluation using a rat parietal bone defect model and did not examine long-term bone maturation or the formation of the interface between PEEK and new bone. Future studies should evaluate the performance of this scaffold in large-animal models and determine the optimal BMP-2 dosage for clinical application by evaluating the BMP-2 side effects, safety profile, and other systemic effects.

## 4. Materials and Methods

### 4.1. Preparation of BMP-2@Gel-PEEK (Figure 1)

A 3D-printed porous PEEK disk (8 mm in diameter and 1 mm in height), composed of PEEK fibers with a diameter of 0.2 mm and a fiber spacing of 0.4 mm, was purchased from Hotty Polymer Co., Ltd., Tokyo, Japan. The mass and theoretically calculated porosity were 46.4 ± 2.0 mg and 44.4%, respectively. After alumina sandblasting for improving water-wettability, the pores of the porous PEEK were filled with aqueous solution of gelatin with an isoelectric point of 9.0 (kindly supplied by Nitta Gelatin Co., Ltd., Osaka, Japan, 100 mg/ml) and the sample was cooled to gel at 4 °C. After freeze-drying, the porous PEEK with gelatin hydrogel was dehydrothermally crosslinked by a vacuum dryer (AVO-250NS; AS ONE Corp., Osaka, Japan) at 160 °C for 72 h to obtain the porous PEEK with gelatin hydrogel (Gel-PEEK). Recombinant human BMP-2 (Qkine Ltd., Cambridge, UK) dissolved in PBS was added to Gel-PEEK, then incubated overnight at 4 °C to obtain Gel-PEEK incorporating BMP-2 (BMP-2@Gel-PEEK).

### 4.2. Characterization of Porous PEEK with Gelatin Hydrogel

The surface of PEEK and Gel-PEEK was observed using a scanning electron microscope (SEM; S-4800, Hitachi High-Tech Corp., Tokyo, Japan). Prior to the SEM observation, a conductive carbon paste (XC-12, Fujikura Kasei Co., Ltd., Tokyo, Japan) was applied to the sides of the sample, and the surface of the sample was coated uniformly with osmium using a hollow-cathode plasma chemical vapor deposition system (HPC-20, Vacuum Device Inc., Mito, Japan). The SEM observation was performed at an accelerating voltage of 5 kV.

To visualize the presence of the gelatin hydrogel inside the porous PEEK, the Gel-PEEK was immersed in an iodine solution (0.1 M, Nacalai Tesque Inc., Kyoto, Japan) of a CT contrast agent for 72 h [45]. Then, the immersed Gel-PEEK was scanned using an X-ray μCT system (Skyscan 1275, Bruker Co., Billerica, MA, USA) at a voltage and current of 60 kV and 85 μA, respectively. After reconstruction, the 3D image was created using CTbox software (version 3.3.0r1403, Bruker Co.).

To investigate the degradation behavior of gelatin hydrogel inside the porous PEEK, the Gel-PEEK (*n* = 3) was immersed in PBS. The supernatant was collected, and the same volume of fresh PBS was added at specified intervals up to 24 h after immersion. Then the immersion solution was changed to PBS with magnesium and calcium ions (PBS (+)) containing collagenase D (25 μg/ml, Roche Diagnostics GmbH, Mannheim, Germany). The supernatant was collected, and the fresh collagenase solution was added at designated intervals. The concentration of collected supernatants containing the degraded gelatin fragments was measured using the bicinchoninic acid (BCA) method (Pierce™ microBCA protein assay kit, Thermo Fisher Scientific Inc., Waltham, MA, USA).

To evaluate the release profile of BMP-2 from Gel-PEEK, the BMP-2@Gel-PEEK (BMP-2 amount: 1 μg, *n* = 3) was soaked in PBS. The supernatants were collected after soaking in PBS and PBS (+) containing collagenase D through procedures similar to those of the degradation test described above. The BMP-2 concentration in each sample was measured using enzyme-linked immunosorbent assay (ELISA) (Quantikine^®^ ELISA kit for BMP-2 (R&D Systems, Inc., Minneapolis, MN, USA).

### 4.3. Animal Experiments

All animal experiments performed in this study were approved by the Animal Care and Use Committee of Osaka Dental University (Approval Nos. 24-05001, 25-02010, and 26-01006) and were compliant with the Osaka Dental University “Regulations on the Care and Handling of Animals.” Sprague–Dawley rats (male, 8 weeks old, Shimizu Laboratory Supplies Co., Ltd., Kyoto, Japan) were maintained under environmentally regulated conditions (temperature: 20 °C, humidity: 50%) with free access to dry food and tap water.

After induction of anesthesia via isoflurane inhalation (FUJIFILM Wako Pure Chemical Corp., Osaka, Japan) until loss of consciousness, a mixed anesthetic solution consisting of medetomidine (0.25 mg/kg; Domitor^®^, Orion Corp., Espoo, Finland), midazolam (3.3 mg/kg; Sandoz K.K., Tokyo, Japan), and butorphanol (4.2 mg/kg; Meiji Animal Health Co., Ltd., Kumamoto, Japan) was injected intraperitoneally. The scalp over the calvarium was shaved using clippers, and remaining hair was removed using a depilatory cream. An incision using a scalpel was then performed on the skin and periosteum of the parietal bone. The skin and periosteum were dissected to expose the bone, and a critical-sized bone defect (9 mm in diameter) was made using a trephine bur (Dentech Corp., Tokyo, Japan). Following the creation of the bone defect, the rats were randomly assigned to four groups (*n* = 5 per group at each time point): (1) only defect without implantation (Control); (2) only PEEK implantation (PEEK); (3) Gel-PEEK implantation; and (4) BMP-2@Gel-PEEK implantation (BMP-2 amount: 5 μg). Finally, the periosteum and skin were sutured. All animals were randomly assigned to groups and handled consistently to minimize variability. Group allocation was known to the investigators during all stages of the experiment, including animal assignment, conduct, outcome assessment, and data analysis. No blinding was performed due to technical limitations. However, to minimize evaluation bias, outcome assessment and data analysis were performed by multiple investigators. Animals (13 rats; 2 (control, 8 wks.), 2 (PEEK, 4 wks.), 4 (PEEK, 8 wks.), 3 (Gel-PEEK, 4 wks.) and 2 (Gel-PEEK, 8 wks.)) determined to have received severe trauma due to technical errors during the creation of bone defects were excluded, and 40 rats were used for the following analyses.

The animals were euthanized via excessive inhalation of isoflurane at 4 and 8 weeks after operation, and their skulls were harvested. After fixation with 20% neutral buffered formalin (Mildform^®^ 20N, Fujifilm Wako Pure Chemical Corp.), the excised skull specimens were shaped using a diamond band saw (BS-3000, EXAKT Advanced Technologies GmbH, Norderstedt, Germany). Bone formation around the defect was evaluated following the μCT analysis and histological staining.

### 4.4. X-Ray μCT Analyses

The shaped skull specimens were scanned using an X-ray μCT system (Skyscan 1275, Bruker Co.) with an aluminum filter at a voltage and current of 60 kV and 85 μA, respectively. Bone formation around the defect was quantitatively analyzed using software (CTAn, version 1.17.4.2, Bruker Co.) to calculate BV/TV, Tb.N, and Tb.Sp. Analyses at all time points were performed under the same thresholding range and region of interest (ROI) definitions. The reconstruction of the obtained μCT images was performed using a voxel size of 30 μm, followed by applying the automatic thresholding algorithm for analysis, which ranged from 70 to 255. Each ROI was set on 81 continuous circular slices with a diameter of 9 mm, encompassing the newly formed bone around the PEEK scaffold while excluding the surrounding native bone tissue. The TV values used in the analysis were obtained by subtracting the theoretically calculated PEEK volume from the TV determined by the software.

### 4.5. Histological Analyses

The shaped skull specimen was sectioned through the center in a coronal plane using a diamond band saw. Half of each specimen was decalcified for H&E and SR staining, while the other half of each specimen was processed without decalcification and used for vK staining.

Decalcification was performed by immersion in a 10% disodium ethylenediaminetetraacetate solution (Muto Pure Chemicals Co. Ltd., Tokyo, Japan) for 14 days. Afterward, the samples were pre-treated with sucrose aqueous solution (FUJIFILM Wako Pure Chemical Corp.) in sequentially increasing concentrations (10%, 20%, and 30%). Cryo-embedding was performed using super cryo-embedding medium (Section-Lab Co. Ltd., Yokohama, Japan). Cryo-sections (10 μm) were prepared using a Leica CM3050S cryostat (Leica Biosystems Nussloch GmbH, Nussloch, Germany) and Cryofilm (Section-Lab Co. Ltd.) according to the Kawamoto method [46].

In H&E staining, the sections were stained with Mayer’s hematoxylin solution. After washing with water and treatment with Tris buffer, the sections were stained with eosin. SR staining was carried out via nuclear staining with Weigert’s iron hematoxylin, differentiation via treatment with 1 N hydrochloric acid in alcohol, washing with water, and collagen staining with SR aqueous solution. The undecalcified frozen sections were used for vK staining. The sections were treated with a silver nitrate solution (5%) under ultraviolet light irradiation, followed by fixation with a 5% sodium thiosulfate solution and nuclear staining with Mayer’s hematoxylin solution. Alcohol dehydration and xylene clearing were performed for sections of H&E, SR, and vK staining before mounting. The stained tissue was observed using the bright-field microscope (BZ-X800, KEYENCE Corp., Osaka, Japan).

### 4.6. Statistical Analysis

Data were presented as mean ± standard deviation. Statistical Analyses were performed using GraphPad Prism 9.5.0 (GraphPad Software, San Diego, CA, USA). One-way analysis of variance was performed for comparisons among groups at each time point, followed by Tukey’s post hoc multiple comparison test. Statistical significance was set at *p* < 0.05.

## Data Availability

The data that support the findings of this study are available from the corresponding author, J.-i.J., upon reasonable request because the dataset includes detailed experimental parameters that are closely related to ongoing and future research.

## Abbreviations

The following abbreviations are used in this manuscript:

BCA	Bicinchoninic acid
BMP-2	Bone morphogenetic protein-2
BV/TV	Ratio of bone volume to tissue volume
3D	Three-dimensional
ELISA	Enzyme-linked immunosorbent assay
H&E	Hematoxylin and eosin
μCT	Micro-computed tomography
PBS	Phosphate-buffered saline
PBS (+)	PBS with magnesium and calcium ions
PEEK	Polyetheretherketone
ROI	Region of interest
SR	Sirius red
Tb.N	Trabecular number
Tb.Sp	Trabecular separation
vK	von Kossa
